# Identification of functional parameters for the classification of older female fallers and prediction of ‘first-time’ fallers

**DOI:** 10.1098/rsif.2014.0353

**Published:** 2014-08-06

**Authors:** N. König, W. R. Taylor, G. Armbrecht, R. Dietzel, N. B. Singh

**Affiliations:** 1 Institute for Biomechanics, ETH, Zürich, Switzerland; 2 Centre for Muscle and Bone Research, Charité—Universitätsmedizin, Berlin, Germany

**Keywords:** falls, functional assessment, gait, balance, force control, principal component analysis

## Abstract

Falls remain a challenge for ageing societies. Strong evidence indicates that a previous fall is the strongest single screening indicator for a subsequent fall and the need for assessing fall risk without accounting for fall history is therefore imperative. Testing in three functional domains (using a total 92 measures) were completed in 84 older women (60–85 years of age), including muscular control, standing balance, and mean and variability of gait. Participants were retrospectively classified as fallers (*n* = 38) or non-fallers (*n* = 42) and additionally in a prospective manner to identify first-time fallers (FTFs) (*n* = 6) within a 12-month follow-up period. Principal component analysis revealed that seven components derived from the 92 functional measures are sufficient to depict the spectrum of functional performance. Inclusion of only three components, related to mean and temporal variability of walking, allowed classification of fallers and non-fallers with a sensitivity and specificity of 74% and 76%, respectively. Furthermore, the results indicate that FTFs show a tendency towards the performance of fallers, even before their first fall occurs. This study suggests that temporal variability and mean spatial parameters of gait are the only functional components among the 92 measures tested that differentiate fallers from non-fallers, and could therefore show efficacy in clinical screening programmes for assessing risk of first-time falling.

## 1. Introduction

The relevance of falls for healthy ageing has been emphasized in numerous studies over the past decades. It is well established that falls are a considerable health threat for ageing populations as well as a serious socio-economic burden for Western societies, with a yearly cost of approximately 1% of the total national healthcare expenditure [1]. Falls commonly lead to fractures of the femoral neck, resulting in hospitalization and a general loss of mobility, but importantly death in some 20% of cases [2]. This makes falls not only the leading cause of mortality after injury in older people [3], but also a serious threat to independent living among the elderly population. Furthermore, with increasing number of older individuals across the world, healthy ageing is a primary focus for researchers and clinicians alike.

Extensive research has been conducted in order to develop screening tools for the identification of individuals with a high risk of falling [4–7], and thus those who will gain the greatest benefit from preventive therapies [8]. However, the large and varied number of risk factors poses a considerable impediment to their success, which has led to wide variety of approaches [9]. Current methods typically range from self-reported questionnaires and clinical assessment of function, through to intensive laboratory evaluation of motion tasks [10–16]. Most commonly used screening tools incorporate a combination of history of falling and clinical mobility assessment (e.g. the Performance Oriented Mobility Assessment (POMA) [6] or the Stratify Screening Test [7]), and have been relatively successful (sensitivity and specificity of 0.75 and 0.80) in assessing fall risk among the elderly [6,7]. However, a large proportion of subjects still cannot be classified accurately. For example, the 23% and 30% levels of positive predictive values of the Stratify and POMA tests, respectively, demonstrate the high false positive rate and therefore the inappropriateness of subject classification [17,18]. Systematic reviews summarizing these screening tools confirm their limited efficacy for faller identification [6,7,17,19].

Irrespective of the tools used for identification of fallers, the best single predictor, as well as the most commonly used factor for fall prediction, is history of falling [14,20]. The relative risk for an older adult to experience a fall is reported to be three times higher for individuals that have fallen previously compared with those that have not [8,21–23]. Although the addition of fall history can improve the accuracy and reliability of identifying individuals with a high fall risk, this parameter is clearly lacking in approaches that attempt to identify subjects with an imminent risk of a first-time fall event. As a result, despite its high predictive power, the parameter ‘history of falls’ cannot be a part of any genuine prospective FTF identification tool. The incorporation of history of falling might also (i) confound the prediction of a faller due to the clear influence of a fall on task performance, (ii) outweigh the benefits of any intervention programme as the best intervention strategies only reduce the risk of falling by a factor of 1, while the risk increases by a factor of 3 after experiencing the first fall [4,5], and (iii) skew the analysis due to the widespread prescription of pharmaceutical therapies. Thus, it would be difficult to interpret whether a particular study outcome is helpful in predicting fall risk or is, in fact, a result of having already experienced a fall event. Although the multi-factorial nature of falls and the lack of a strong fall-related ‘biomarker’ make identification of FTFs a burgeoning challenge, targeted prevention programmes can only be effectively implemented after the successful prospective identification of FTFs.

Although falls are considered a multi-factorial problem where environmental, intrinsic and external factors are all possible triggers for the occurrence of a fall [8,20], functional indices of muscle strength, gait and balance are all known to play critical roles [8,20,21]. However, rehabilitation programmes focused on improving their functional ability have not led to the expected reduction in falls, possibly due to how these physiological aspects have been reported or interpreted. For example, while muscle strength is an important physiological parameter, most activities of daily living are performed at submaximal levels [24]. Similarly, it has been reported that inter-stride variations during continuous or repetitive task performance (e.g. gait variability) might be better suited to capturing the dynamics of human walking rather than the summary measures of walking speed, cadence or even step length [25,26]. In fact, fallers exhibit larger levels of intra-task variability than their healthy counterparts during balance and gait [27,28], and prospective studies have also confirmed that extreme levels of variability might be the cause and not the consequence of falls [26,29–31], and careful assessment of these factors might therefore allow improved identification of subjects at risk of first-time falling [26,29–31]. However, until now, the relative contribution of measures of variability towards identifying fallers from non-fallers remains unknown, but particularly whether these intrinsic factors aid in the identification of FTFs.

Through extracting the principal components from multiple functional measures based on gait, gait variability, balance, lower extremity strength and force control ability, in cohorts of fallers and non-fallers, this study aimed to establish which components are able to best distinguish between fallers and non-fallers in a retrospective study design. Furthermore, the efficacy of these components to identify FTFs before the onset of an actual first-time fall event was targeted in a prospective study design.

## 2. Material and methods

### 2.1. Participants

Within a larger study investigating multiple aspects of osteoporosis in older women (EU VPHOP FP7–223864), we recruited 90 older female participants (60–85 years of age) from the local community (by public announcement in local hospitals, physiotherapy practice and gyms), that were identified as ‘faller’ (F) or ‘non-faller’ (NF) based on the question ‘Have you experienced a fall within the previous 12 months?’ Recruitment was conducted in order to ensure that two homogeneous groups were produced. This study focused on assessing the intrinsic factors associated with falling. Exclusion criteria were

— BMI < 18 or BMI > 33,
— alcoholism (more than 3 units/day),
— type 1 diabetes, cardiac infarct, chronic hepatitis, celiac and mal-absorption diseases, rheumatoid arthritis, hyperparathyroidism, hyperthyroidism, cancer,
— treated for more than three months or under treatment with oral corticosteroids,
— subjects with neurological diseases affecting the neuromuscular system such as: Parkinson's disease, muscular dystrophy, ankylosing spondylitis, myopathies, myasthenia, cerebral trauma, stroke, peripheral neurosystem diseases,
— fractures/osteosyntheses/degenerative changes that might cause invalid results in DXA measurements,
— femur fracture or total hip replacement (less than six months),
— subjects who are unable to follow the examinations of the study protocol or unable to walk 10 m without a walking aid and
— participation in another study at the same time.

Prior to testing, subjects were classified retrospectively as ‘fallers’ (*n*_F_ = 42) or ‘non-fallers’ (*n*_NF_ = 48), based on the question ‘if they had experienced a fall within the previous 12 months’. Of these 90 older women, only 84 subjects, including 42 fallers and 42 non-fallers completed all laboratory-based functional assessments (table 1). All participants provided written informed consent and the experiments were approved by the local ethics committee. Furthermore, of the 42 fallers, four were removed from the analysis, since their fall event occurred during sports or strenuous activities such as skiing or cycling and was therefore considered not to be consistent with a typical uncoordinated fall event. All 80 remaining participants, consisting of 38 fallers and 42 non-fallers, completed a series of functional tests as described below.

**Table 1.** Anthropometrics and self-reported daily physical activity, alcohol consumption and medical intake for subjects of NF and F group.

	NF	F
age (±s.d.), years weight (±s.d.), kg height (±s.d.), cm	68.9 (±4.5) 68.7 (±10.3) 161.8 (±6.1)	69.2 (±4.8) 69.9 (±9.9) 162.9 (±6.9)
visual impairment pain in last 7 days	32 30	37 27
diabetes	3	2
average daily activity		
light	10	9
moderate	20	22
heavy	12	11
alcohol frequency		
not at all	6	7
less than 1/week	17	14
1–2 days/week	10	10
3–4 days/week	3	9
5–6 days/week	4	1
every day	2	1
medication		
no	6	4
1 drug	18	19
2 or more drugs	18	19

**Figure 1.**
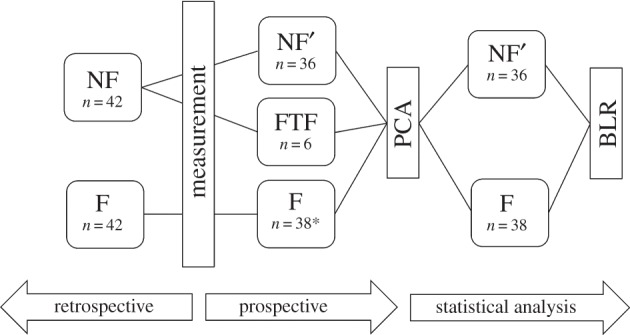
Schematic of the statistical approach, showing the combined retrospective and prospective classifications. NF: non-faller; F: faller; FTF: first-time-faller; NF’: non-faller without FTFs. Asterisk (*) denotes four subjects were removed from the faller cohort because of extraordinary fall circumstances.

In addition, fall monitoring was conducted via a postal questionnaire 12 months after the laboratory measurements in order to identify previous NFs who underwent a first-time fall within the 12 month follow-up period (‘FTFs’). Here, questionnaires were sent together with prepaid envelopes for their return. In the questionnaires, subjects were asked if they had experienced a fall within the previous 12 months, if they experienced a loss of consciousness or vertigo before the fall and to provide a detailed description about the fall circumstances. The NF group without the FTFs was then re-classified NF’ (figure 1).

### 2.2. Experimental design and procedures

Subjects underwent three separate measurement sessions aimed to examine repetitive or continuous performance of force production, postural sway and gait. All tests were conducted on the same day and each participant was provided sufficient time and practice in order to familiarize themselves with each task.

#### 2.2.1. Strength measurements

In order to assess muscular strength, maximum voluntary isometric contractions (MVICs) were assessed in the knee extensors and ankle plantarflexors using a dynamometer (Biodex 3 Pro, Biodex Medical Systems Inc., USA). Participants were seated in a standardized position [32]. Knee extension measurements were performed with the right knee flexed at 90°, while for ankle measurements, the knee was fully extended with 10° of plantarflexion at the ankle. Individuals were requested to push ‘as hard as they could’ against the attachments for a period of 10 s, while receiving verbal encouragement. MVICs for both the knee extensors and ankle plantarflexors were each measured three times with a minimum of 30 s pause between contractions [33]. The single greatest value from the three contractions for the knee as well as the ankle was then used as the respective MVIC.

#### 2.2.2. Muscular control

In order to assess muscular control, the quality of continuous force production was assessed using the same experimental set-up as described above. Here, an objective or target torque (TT) of either 15, 20 or a ramped 15–20% of the MVIC level was provided visually on a digital monitor. The active torque applied by the participant was then displayed as a real-time visual feedback at 10 Hz, which overlaid the TT. Participants were instructed to match the torque level ‘as best they could’ for the duration of the 15 s test by performing isometric knee extension or ankle plantarflexion, respectively. Participants were provided four to five practice test repetitions to familiarize themselves with the experimental procedures. The presentation order of the signals was randomized, with all TTs (i.e. constant 15%, constant 20% and ramp of the 15–20% MVIC) presented a minimum of three times. The error, or fluctuations within the force output signal, was then considered a measure of muscular control.

#### 2.2.3. Quiet standing

The older individuals were requested to perform quiet standing tasks, in order to assess their postural sway. Subjects performed trials with eyes open (QEO) and closed (QEC) for a duration of 30 s. In the QEO condition, participants focused on a visual target, positioned at eye level on the wall, approximately 3 m in front of them, and were instructed to stand as still as possible, while barefoot with their hands by their sides. The participants’ feet were positioned on two separate force platforms (AMTI OR6–7–1000, Watertown, MA, USA). Each subject was provided a minimum of 60 s practice in order to familiarize themselves with the test, before performing three repetitions of each task. At least 1 min relaxation was provided between each sway test. The tri-axial force data during standing tasks were recorded at 120 Hz in order to allow determination of measures of the centre of pressure (CoP).

#### 2.2.4. Gait analysis

In order to assess numerous functional parameters of gait, participants were requested to walk barefoot along a 10 m straight walkway, at their preferred walking speed, with recording beginning after at least three practice walks. A minimum of six walks were then measured for the determination of mean measures of gait, as well as gait variability. Three-dimensional kinematics of both feet were captured using a 10-camera motion capture system (Vicon, OMG Ltd, Oxford, UK), where eight reflective markers (14 mm) were attached to the skin at different bony landmarks: tuber calcanei (heel), caput ossis metatarsale I (first metatarsus), caput ossis metatarsale V (fifth metatarusus) and at the base of the os metatarsale II and III (at the base of the second and the third metatarsus). The first and last strides from each walk were removed to avoid transients, leaving a total of approximately 30–40 strides for analysis.

### 2.3. Data analysis

#### 2.3.1. Muscular control

All torque measurements were collected using Labview (Labview 8.6, National Instruments, Inc., USA). From each trial, the first 7 s and the last 2 s of torque output were removed to avoid any transients during initiation or termination of the trials. All data were then low-pass filtered (fourth-order, zero-phase lag, Butterworth, 25 Hz cut-off frequency). In order to assess force fluctuations, both mean and standard deviation (SD) of the force production signal were evaluated [32]. In addition, the coefficient of variation (CV) of the force produced was calculated as the ratio of the SD to the mean of the force output for each type of muscular control test, and for each joint.

#### 2.3.2. Quiet standing

The obtained CoP time-series [34] was pre-processed by firstly removing the initial and final 2 s of data to avoid boundary effects, and then low-pass filtering (Butterworth, second-order, bi-directional, 5 Hz cut-off frequency). From the CoP time-series, the mean (Mn-DIST) and root mean square (RMS) distance, area (AREA), elliptical area (EA), mean velocity (Mn-VEL) and mean sway frequency (Mn-FREQ) were calculated for the entire datasets, as well as for both the anterior–posterior (AP) and medio-lateral (ML) directions individually [28,35].

#### 2.3.3. Gait analysis

The trajectories of both heel markers, together with the markers at the base of the second and the third metatarsus, were used to extract stride time information. After low-pass filtering (Butterworth, fourth-order, bi-directional, 25 Hz cut-off frequency), heel strikes were identified using a foot velocity algorithm [36]. Two consecutive heel strikes defined a single stride. Stride time was calculated as the time elapsed between two consecutive heel strikes, while the distance between heel strikes in the direction of walking progression provided the stride length. Cadence (CAD) was calculated based on the stride time information, while double support time (DST) was determined as the time interval during which both feet were in contact with the ground. The projected distance in the medio-lateral direction from successive heel strikes of opposite feet was evaluated to provide step width (SW). The maximum foot clearance (MaxFC) was calculated as the maximum vertical distance between the foot and the ground, while minimum foot clearance (MinFC) represented the minimum vertical distance between the foot and the ground during the mid-swing phase [37,38]. Walking performance and its variability was assessed using the mean, SD and CV of the described gait parameters. To represent the concept of gait variability, both SD as an absolute measure and CV as a relative measure of gait variability were included in analysis, providing different predictive powers for the identification of F and NF. As the clear aim of this study was to avoid subjective pre-selection of parameters, we included all 92 parameters and then applied principal component analysis (PCA) to objectively avoid redundant information. All the calculations were conducted using Matlab (R2011b, MathWorks, USA).

### 2.4. Statistical analyses

#### 2.4.1. Principal component analysis

In total, 92 measures (electronic supplementary material, table S1) were used to quantify the continuous performance of force production, postural sway and gait from the 80 participants (producing a matrix of 80 × 92). All measures were converted into standardized *Z*-scores, thus providing effective management of any missing data. Here, missing values were simply replaced with the mean of the sample (i.e. zero) and the actual measurement values were interpreted in relation to the respective deviations from the mean.

In order to preserve the intrinsic features of task performance, as well as to reduce the effective dimensions of the entire dataset, factor analysis (FA) was performed using the FACTOR procedure (SPSS v. 20, IBM, USA). This correlation analysis method was applied to extract the components prior to undertaking the PCA using the ‘VARIMAX’ rotation procedure. The Kaiser criterion (i.e. components that had Eigenvalues greater than one), was used to extract the appropriate number of components [39]. In addition, two more criteria were applied to ensure the consistency of the original measures: (i) measures with a measure of sampling adequacy less than 0.5 and (ii) measures that caused complex structure, i.e. were loaded (correlated with *r* > 0.4) by two or more components, were removed from the analysis in an iterative process to ensure appropriate parameter selection [39]. The component scores obtained by the PCA were then used for all further statistical analyses.

#### 2.4.2. Binary logistic regression

A binary logistic regression was conducted to assess the ability of measures derived from activities of daily living to predict the ‘intrinsic’ susceptibility of elderly towards experiencing a fall. The dependent variable, fall_experience_, was dichotomous, with those that had experienced a fall in the previous 12 months (fall_experience_ = 1) or non-fallers (fall_experience_ = 0). The independent variables in the analysis were the extracted component scores, obtained from the PCA. Finally, Hosmer Lemeshow test was conducted in order to test the goodness of fit of the logistic regression.

The significance level for all analyses was set at *p* < 0.05 and all statistical analyses were conducted using SPSS v. 20 (IBM, USA).

## 3. Results

### 3.1. Retrospective classification of fall status

#### 3.1.1. Principal components

A total of six iterations were required within the PCA to reach measure of sampling adequacy levels above 0.5, as well as being devoid of any complex structure, after which seven components were obtained, representing 90% of the total variance of the entire dataset (Kaiser–Meier–Olkin = 0.714). These seven components were loaded with 31 measures based upon the extracted and weighted coefficients (table 2). As a result, the first component represented standing task performance during closed eyes condition and was interpreted as *static balance*. Similarly, components 2 and 3 represented the mean temporal and spatial characteristics of gait, respectively, and interpreted as *temporal gait* and *spatial gait*. Components 4 and 5 contained information regarding temporal variability of gait (inter-cycle variations) from the right and left feet, respectively, and were interpreted as *temporal variability right* and *left*. The sixth component represented spatial variability (inter-cycle variations) during walking—*spatial variability*. Finally, the last component represented the inter-cycle variability of double support stance time and is termed *dynamic balance*.

**Table 2.** Compilation of characteristic components of function with communalities and the variance explained by the component. Only those coefficients that had a considerable influence (more than 0.5) on the component are presented in order to ease interpretation of the obtained components. The first two letters of each functional parameter indicate the test domain: QS, quiet standing; GA, gait analysis. The first two letters after the colon indicate either: Mn, mean; SD, standard deviation; CV, coefficient of variation. The remaining acronyms indicate the actual measure as described in the methods section. VEL, velocity of sway; AREA, area of sway; FREQ, frequency of sway; EA, elliptical area of sway; AP, anterior–posterior direction; ML, medio-lateral direction (if not applicable, the total sway path was analysed); QEC, quiet standing eyes closed; CAD, cadence; Str-T, stride time; Stn-T, stance time; DST, double support time; SW, swing time; Str-Len, stride length; Stp-Len, step length; MaxFC, maximum foot clearance; R, right foot; L, left foot.

functional measures	*static balance*	*temp. gait*	*spatial gait*	*temp. variab., right*	*temp. variab., left*	*spatial variab.*	*dynamic balance*	communalities
QS: Mn-VEL-QEC	0.988							0.989
QS: Mn-VEL-AP-QEC	0.985							0.984
QS: Mn-VEL-ML-QEC	0.985							0.985
QS: Mn-AREA-QEC	0.959							0.952
QS: Mn-FREQ-AP-QEC	0.955							0.921
QS: Mn-EA-QEC	0.954							0.934
QS: Mn-FREQ-QEC	0.952							0.914
QS: Mn-FREQ-ML-QEC	0.822							0.729
GA: Mn-CAD		−0.937						0.941
GA: Mn-Str-T-L		0.926						0.981
GA: Mn-Stn-T-L		0.871						0.968
GA: Mn-DST-L		0.773						0.846
GA: Mn-Sw-T-R		0.744						0.709
GA: Mn-DST-R		0.728						0.840
GA: Mn-Str-Len-R			0.920					0.935
GA: Mn-Str-Len-L			0.918					0.929
GA: Mn-Stp-Len			0.902					0.913
GA: Mn-MaxFC-R			0.866					0.777
GA: Mn-MaxFC-L			0.856					0.765
GA: CV-Stn-T-R				0.988				0.993
GA: CV-Str-T-R				0.988				0.997
GA: SD-Stn-T-R				0.970				0.991
GA: SD-Str-T-R				0.969				0.993
GA: CV-Str-T-L					0.991			0.992
GA: SD-Str-T-L					0.983			0.987
GA: SD-Stn-T-L					0.979			0.987
GA: SD-Str-Len-L						0.949		0.956
GA: CV-Str-Len-L						0.938		0.961
GA: CV-DST-L							0.914	0.875
GA: SD-DST-L							0.858	0.841
GA: SD-Sw-T-L							0.572	0.624
Variance explained by the component (%)	23	15	14	13	11	7	7	

#### 3.1.2. Binary logistic regression

The binary logistic regression procedure revealed a significant relationship between components and fall_experience_ (Nagelkerke coefficient of determination, *R*^2^ = 0.26; Homser Lemeshow goodness of fit *p* = 0.44; table 3). Only three of the seven components (*spatial gait*, *temporal variability left* and *temporal variability right*—in order of significance) exhibited high importance (*p* < 0.1) and were further used to predict fall_experience_ (electronic supplementary material, table S2). The regression resulted in the following equation derivation (table 3):3.1
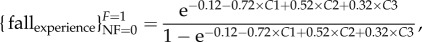
where fall_experience_ is the dichotomous-dependent variable with faller group F = 1 and non-faller group NF = 0. *C1*–*C3* are the independent variables extracted from the functional parameters via the PCA (i.e.: *C1* = *spatial gait; C2* = *temporal variability left; C3* = *temporal variability right*).

**Table 3.** Results of binary logistic regression. *Spatial gait, temporal variability right* and *left* enter the regression equation at an alpha level of *p* < 0.1.

	*β*	s.e.	Wald	*p*	exp(*β*)
*static balance*	1.4	0.97	2.1	0.15	4.1
*temporal gait*	−0.01	0.27	0.002	0.96	0.99
*spatial gait*	**−0.94**	**0.34**	**7.90**	**0.005**	**0.39**
*temporal variability, right*	**0.45**	**0.27**	**2.82**	**0.093**	**1.57**
*temporal variability, left*	**0.64**	**0.28**	**5.27**	**0.022**	**1.90**
*spatial variability*	0.17	0.26	0.41	0.52	1.18
*dynamic balance*	0.21	0.25	0.71	0.40	1.23
constant	0.20	0.26	0.003	0.96	1.02

**Figure 2.**
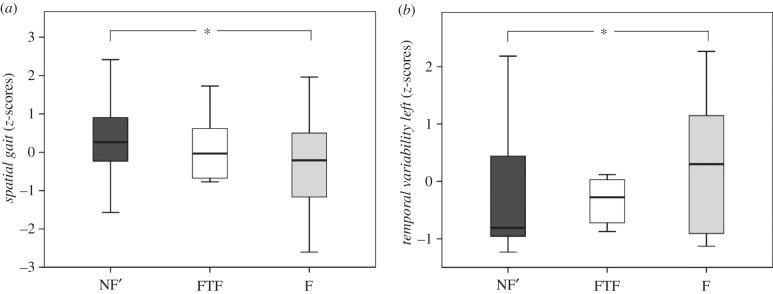
Scores for *spatial gait* (*a*) and *temporal variability, left* (*b*) for the three patient groups NF’, FTF and F. While results for FTF are displayed, they were not considered in the regression analysis due to the unequal sample size. Asterisk (*) donates significance at *p* = 0.05.

The levels of sensitivity and specificity achieved by the model for identifying fallers were 74% and 76%, respectively (table 4). Furthermore, positive (PPV) and negative (NPV) predictive values were 74% and 76%, respectively. The binary logistic regression revealed that fallers had significantly reduced *spatial gait* scores and significantly increased *temporal variability left* scores (figure 2).

**Table 4.** Classification of retrospectively identified fallers and non-fallers, showing sensitivity, and specificity, as well as positive (PPV) and negative predictive value (NPV) in %. FTFs were considered to be non-fallers (i.e. at the measurement time point). From the faller cohort, four subjects were removed from the analysis because of extraordinary fall circumstances.

		observed		
		F	NF	
predicted	F	28	10	PPV 74
	NF	10	32	NPV 76
		sensitivity	specificity	
		74	76

### 3.2. Prospective assessment of fall risk

The follow-up postal questionnaire revealed a 100% response rate with 10 individuals (of the 80 included in this study) reporting at least one fall within the 12-month follow-up period. Six of these 10 individuals were FTF (previously classified as NF; 68.3 ± 4.9 years, 63.9 ± 4.4 kg, 160.9 ± 8.7 cm) and four were repeat fallers (previously already classified as F). Post hoc power analysis revealed that 13–20 subjects would be required to reach statistical power of 80%. Since only six subjects were included within this group, only limited conclusions can be drawn from this sample. Although no statistical tests could be undertaken with the FTF group in a prospective manner due to the small and uneven group sizes, FTFs had lower cumulative *spatial gait* scores and greater cumulative *temporal variability* scores compared with the NF’ cohort, and approaching those of the F cohort (figure 2).

## 4. Discussion

With an increasing proportion of elderly worldwide, falls among older individuals already contribute to over 1% of annual total healthcare expenditure [1]. While a variety of tools, questionnaires and assessment methodologies exist to identify subjects at high risk of falling, these have generally been unsatisfactory, primarily because falls are a multi-factorial phenomenon. An individual's fall history remains the single best predictor of fall risk, but its use is inevitably excluded in the identification of subjects at risk of falling prior to their first fall event. Therefore, this study aimed to extract multiple measures of task performance that show potential as biomarkers for evaluating fall risk and particularly that allow identification of those subjects that are at risk of experiencing a first-time fall. The results of the study suggest that seven components are able to capture the most essential characteristics that differentiate fallers from non-fallers, and that mean stride and step length, as well as inter-cycle temporal variability, are sufficient for predicting the risk of falling with 74% and 76% sensitivity and specificity, respectively, and an overall *retrospective* post hoc statistical power of 95%.

The use of PCA allowed (i) an effective combination of functional measures across all domains, while removing redundancies in the dataset and (ii) an investigation of common components by grouping subsets of parameters with high correlation while remaining relatively independent of other parameters [39]. The seven components obtained from the PCA, which were extracted from a total of 92 functional measures, displayed a high reliability, as reflected by the Kaiser–Meier–Olkin test (0.714, where values > 0.6 are considered good) [39]. This suggests good interpretability of the extracted components while also reflecting a comprehensive representation of the functional performance among subjects. Interestingly, measures of muscular control (i.e. inaccuracy and fluctuations during force production tasks) had to be excluded during the PCA owing to the complex nature of their loading across different components (possessed weighted correlation coefficients larger than 0.5). This behaviour of muscular control at submaximal levels is not surprising, as previous studies have demonstrated a relationship between force fluctuations and task variability during both standing as well as walking tasks [40]. Similarly, the parameters capturing maximum isometric strength from ankle plantarflexors and knee extensors also exhibited a complex structure and had extremely low levels of sampling adequacy (MSA < 0.5). The lower levels of sampling adequacy, especially for knee extensors (MSA = 0.12) in this study, suggest that inclusion of these parameters in combination with other functional domains of quiet standing and walking does not provide any unique metric for task performance. Similarly, low levels of measure of sampling adequacy were also obtained from quiet standing with eyes open. This suggests that incorporating standing with eyes open, together with walking tasks and standing with eyes closed, might be redundant for identifying motor-related deficits. One possible explanation is that during both standing and walking tasks, the primary role of the human sensorimotor system is to maintain the centre-of-mass within the base-of-support [41], and this is clearly more challenging during standing with eyes closed compared to standing with eyes open. Moreover, walking is the most frequent activity of daily living, but counterintuitively, older individuals who walk more indirectly increase their susceptibility to falling [42–44], possible due to the longer periods on their feet.

Standing with eyes closed provided both a unique set of information and formed the first of the seven components (*static balance*). As PCA provides an un-biased extraction of parameters, the ranking of the components was not relevant, but was rather based on the amount of variation across the sample of subjects. The large variation captured by *static balance* (23%; table 2) probably pertains to the complexity of the task (standing with eyes closed) rather than the predictive power of this component on risk of falling, which was revealed by conducting the binary logistic regression. All the other components represented performance during walking. Although there may have be some redundancy in our choice of the gait variables calculated, the PCA was useful in condensing these data into useful and interpretable factors. Important aspects highlighted by the results pertaining to the extraction of components via PCA within this study were that:

— components summarizing mean parameters of walking (*temporal gait* and *spatial gait*) were not associated with those summarizing inter-cycle variability of walking (*temporal variability right*, *temporal variability left, spatial variability and dynamic balance*),
— components summarizing spatial aspects of walking (*spatial gait* and *spatial variability*) were unique to those representing temporal aspects of walking (*temporal gait*, *temporal variability right* and *temporal variability left*),
— parameters summarizing mean levels of walking captured from left and right limb kinematics were non-unique (*temporal gait* and *spatial gait*). However, parameters that summarized inter-cycle variability were specific to the right and left limbs (*temporal variability right* and *temporal variability left*).

These results highlight the importance of assessing walking performance, particularly mean parameters of stride and step length, but these measures alone are insufficient to capture the dynamics of the sensorimotor system [25,26,45]. Importantly, this study clearly indicates that these measures need to also be complemented by considering inter-cycle variability in temporal parameters during continuous walking. These results suggest that spatial and temporal aspects of walking might be governed through different control mechanisms within the human sensorimotor system [46–48].

While the levels of both sensitivity and specificity were around 75% in this study, not remarkably higher compared with other studies, the positive and negative predictive rates reported here were higher than values reported elsewhere in the literature [7,10,17,23,30], which include standard approaches used in the clinic. This indicates that the comprehensive combination of functional measures considered here was able to predict the risk of falls in older individuals with moderate to low false positive and negative rates. The reduced levels of false positives and negatives could be a result of the use of a wide spectrum of measures that directly assessed the performance of normal functional activities of daily living. Furthermore, in contrast to other fall risk screening tools that were based on pre-selected parameters, this study rather aimed to collect a comprehensive range of functional data, and then remove the redundant information using an unbiased principal component approach [49].

The component *spatial gait* contains mean stride length, mean step length and maximum foot clearance. A direct comparison of this component revealed that fallers walked with shorter steps and exhibited reduced foot clearance compared to their non-falling counterparts (figure 2). While reduced step length and height have previously been associated with fall risk [50,51], it is still controversially discussed whether such changes are due to fear of falling or to an increased requirement for stability during walking [52,53]. A direct comparison of the *temporal variability* component, including absolute (SD) and relative (CV) temporal inter-cycle variability of stride and stance time, revealed increased levels of temporal gait variability among fallers compared with non-fallers (figure 2), which is consistent with the literature [30,44,51,54]. Furthermore, recent investigations have shown that increased levels of inter-cycle variability during walking could move the centre-of-mass closer to the limits of stability (base-of-support) [54,55], which would require the human sensorimotor system to generate joint torques to maintain the boundary constraints. In this study, increased gait variability was accompanied by reduced stride and step lengths in the faller cohort, thus supporting the concept that reduced stride and step length is used as a compensation mechanism for maintaining stability during walking.

The secondary aim of this study was to assess task performance in individuals who experienced a first-time fall with an overriding goal of predicting the risk of such an event in older individuals. The rational for such a goal stems from the overwhelming evidence that older individuals continue to fall after experiencing their first fall, even after participating in fall prevention programmes [4,5]. Consequently, there is a critical requirement to prevent falling before the first fall event, but the usage of fall history as an identification parameter becomes redundant in this case, thus indicating the use of intrinsic functional control metrics to complement clinical questionnaires. Unfortunately, owing to the small sample size for the FTF group, no far-reaching conclusions could be drawn on the efficacy of functional parameters for identifying FTF. However, this group was clearly positioned between the non-fallers (NF’ after excluding the FTF) and fallers, indicating that this group had a tendency towards decreased mean spatial gait and increased temporal gait variability, as seen in fallers (figure 2). A post-hoc sample size estimation using power analysis based on a set of parameters (e.g. Mn-MaxFC-R, Mn-MaxFC-L, SD-Stn-T-L, etc.; electronic supplementary material, table S1) revealed a required sample size of 13–20 FTF subjects before reliable conclusions can be drawn with a statistical power of 80%, suggesting future studies should include at least 91 non-fallers with similar demographics. A further limitation of this study was the extensive exclusion criteria, which resulted from the subject recruitment within a larger study. Here, certain pathologies that are typical for the elderly population were excluded. However, it was the clear aim of this study to improve our understanding of the intrinsic control mechanisms and the role of functional measures on falling, rather than external factors such as medication or alcohol that were controlled for and were homogeneous between groups. A general issue that needs to be considered in self-reported fall assessments is under-reporting, which is known to result in inaccuracies of approximately 15–30% [56,57]. It is quite possible that under-reporting resulted in the low percentage of 12.5% of prospective falls observed in this study, which is below what has been reported in similar studies [2,3]. Furthermore, tactile sensation and dementia were not assessed in this trial, which could play a role on gait and balance. However, although it was not possible to identify the aetiology underlying each fall, such comorbidities could be considered to be encompassed within the wide-ranging spectrum of functional domains (including strength, gait, balance, etc.) that were assessed within this study. Since gait was the dominant discriminatory domain, new technologies such as body-worn inertial sensors that are able to capture measures of gait and its variability with sufficient sensitivity [58] might allow rapid screening of larger patient numbers in clinical settings without the need for laboratory-based investigations.

The results of this study suggest that task-related deficits such as isometric muscle strength and standing balance with eyes open might be redundant compared to parameters of walking. While further investigation towards quantifying the efficacy of functional parameters for predicting FTFs in larger population-based studies is indeed required, the research conducted here has demonstrated that mean parameters of gait and their variability are key components for assessing motor-related deficits in the elderly, and could well aid in clinical screening programmes for identifying FTFs.

## Supplementary Material

Complete list of functional measures

## Supplementary Material

Component coefficients, group means and standard deviation for included functional measures
