# Supplementary figures and images for: Identification of functional parameters for the classification of older female fallers and prediction of ‘first-time’ fallers
Source: J R Soc Interface. 2014 Aug 6;11(97):20140353. doi: 10.1098/rsif.2014.0353 (PMC4208368; doi:10.1098/rsif.2014.0353)

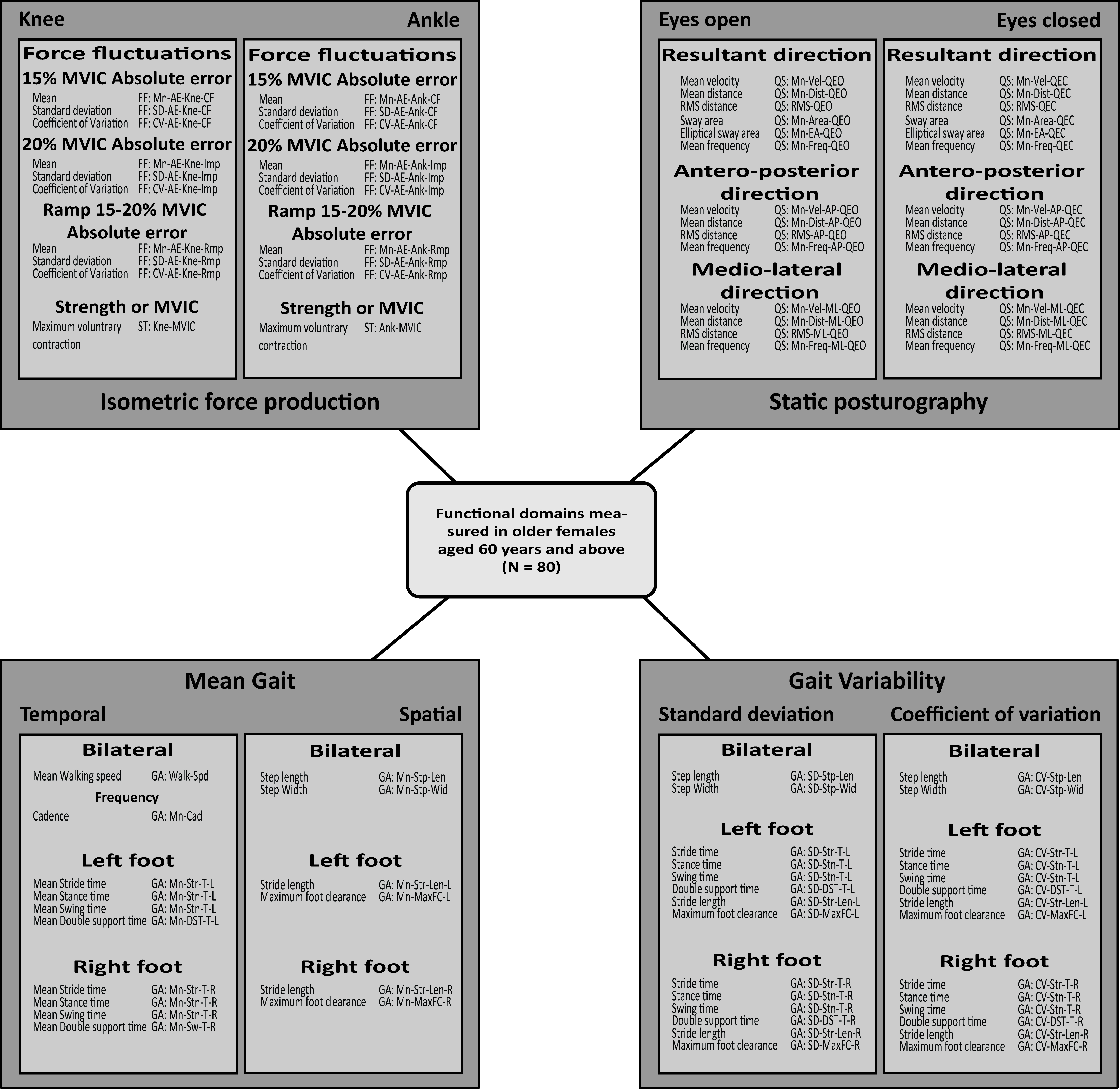

Supplement: Complete list of functional measures [file rsif20140353supp1.jpg]
