# Supplementary material for: Identification of functional parameters for the classification of older female fallers and prediction of ‘first-time’ fallers
Source: J R Soc Interface. 2014 Aug 6;11(97):20140353. doi: 10.1098/rsif.2014.0353 (PMC4208368; doi:10.1098/rsif.2014.0353)
Supplement: Component coefficients, group means and standard deviation for included functional measures [file rsif20140353supp2.doc]

ESM Table 2: Component coefficients, group means and standard deviation for included functional measures.

| **Components** | **Functional Measures** | **Component coefficients** | **NF** | **F** |
| --- | --- | --- | --- | --- |
| ***Static balance*** | QS: Mn-VEL-QEC | 0.988 | -0.12 (0.13) | 0.14 (1.48) |
|  | QS: Mn-VEL-AP-QEC | 0.985 | -0.12 (0.12) | 0.15 (1.48) |
|  | QS: Mn-VEL-ML-QEC | 0.985 | -0.12 (1.35) | 0.14 (1.48) |
|  | QS: Mn-AREA-QEC | 0.959 | -0.11 (0.02) | 0.14 (1.49) |
|  | QS: Mn-FREQ-AP-QEC | 0.955 | -0.07 (0.40) | 0.08 (1.43) |
|  | QS: Mn-EA-QEC | 0.954 | -0.16 (0.19) | 0.19 (1.46) |
|  | QS: Mn-FREQ-QEC | 0.952 | -0.04 (0.44) | 0.06 (1.41) |
|  | QS: Mn-FREQ-ML-QEC | 0.822 | 0.08 (0.71) | -0.12 (1.27) |
| ***Temporal gait*** | GA: Mn-CAD | -0.937 | 0.07 (0.78) | 0.04 (1.04) |
|  | GA: Mn-Str-T-L | 0.926 | -0.12 (0.83) | 0.02 (1.08) |
|  | GA: Mn-Stn-T-L | 0.871 | -0.10 (0.87) | 0.01 (1.06) |
|  | GA: Mn-DST-L | 0.773 | -0.21 (0.39) | 0.04 (0.66) |
|  | GA: Mn-Sw-T-R | 0.744 | -0.08 (0.91) | 0.15 (1.02) |
|  | GA: Mn-DST-R | 0.728 | -0.19 (0.39) | 0.02 (0.56) |
| ***Spatial gait*** | GA: Mn-Str-Len-R | 0.920 | 0.14 (0.75) | -0.33 (0.88) |
|  | GA: Mn-Str-Len-L | 0.918 | 0.15 (0.74) | -0.34 (0.90) |
|  | GA: Mn-Stp-Len | 0.902 | 0.11 (0.68) | -0.31 (0.79) |
|  | GA: Mn-MaxFC-R | 0.866 | 0.27 (0.83) | -0.35 (1.06) |
|  | GA: Mn-MaxFC-L | 0.856 | 0.29 (0.86) | -0.33 (1.02) |
| ***Temporal variability, right*** | GA: CV-Stn-T-R | 0.988 | -0.19 (1.02) | 0.13 (0.88) |
|  | GA: CV-Str-T-R | 0.988 | -0.21 (0.91) | 0.10 (0.81) |
|  | GA: SD-Stn-T-R | 0.970 | -0.21 (0.93) | 0.12 (0.80) |
|  | GA: SD-Str-T-R | 0.969 | -0.22 (0.80) | 0.09 (0.73) |
|  | GA: CV-Str-T-L | 0.991 | -0.20 (0.82) | 0.17 (0.84) |
| ***Temporal variability, left*** | GA: SD-Str-T-L | 0.983 | -0.21 (0.70) | 0.14 (0.77) |
|  | GA: SD-Stn-T-L | 0.979 | -0.23 (0.74) | 0.18 (0.87) |
| ***Spatial variability*** | GA: SD-Str-Len-L | 0.949 | -0.13 (0.12) | -0.08 (0.15) |
|  | GA: CV-Str-Len-L | 0.938 | -0.15 (0.19) | -0.05 (0.30) |
| ***Dynamic balance*** | GA: CV-DST-L | 0.914 | -0.14 (0.33) | -0.05 (0.22) |
|  | GA: SD-DST-L | 0.858 | -0.12 (0.09) | -0.09 (0.08) |
|  | GA: SD-Sw-T-L | 0.572 | -0.07 (0.97) | 0.10 (1.07) |

List of included measures and derived components. The component coefficients and means (SD) for both groups (NF and F) based on z-scores are presented.
